# Cytochrome P450 1A2 Is Incapable of Oxidizing Bilirubin Under Physiological Conditions

**DOI:** 10.3389/fphar.2019.01220

**Published:** 2019-10-18

**Authors:** Xinyi Li, Dongzhen Yu, Huiqun Jie, Huiqun Zhou, Haibo Ye, Guo Ma, Lili Wan, Chunyan Li, Haibo Shi, Shankai Yin

**Affiliations:** ^1^Department of Otorhinolaryngology-Head and Neck Surgery, Shanghai Jiao Tong University Affiliated Sixth People’s Hospital, Shanghai, China; ^2^Shanghai Key Laboratory of Sleep Disordered Breathing, Shanghai, China; ^3^Department of Clinical Pharmacy, School of Pharmacy, Fudan University, Shanghai, China; ^4^Department of Pharmacy, Shanghai Jiao Tong University Affiliated Sixth People’s Hospital, Shanghai, China

**Keywords:** bilirubin, cytochrome P450, brain, metabolism, LC/MS-MS

## Abstract

**Background:** Bilirubin (BR) is metabolized mainly by uridine diphosphate (UDP)-glucuronosyltransferase 1A1 (UGT1A1) through glucuronidation in the liver. Some studies have shown that several subtypes of cytochrome P450 (CYP) enzymes, including CYP1A2, are upregulated by inducers and proposed to be alternative BR degradation enzymes. However, no information is available on the BR degradation ability of CYP in normal rats without manipulation by CYP inducers.

**Methods:** Quantitative real-time polymerase chain reaction (QRT-PCR), western blot, immunofluorescence, and confocal microscopy were used to find expression of CYP1A2 in the brain and the liver. BR metabolites in microsomal fractions during development were examined by high-performance liquid chromatography/electrospray ionization tandem mass spectrometry (LC-MS/MS).

**Results:** In the present study, we observed that CYP1A2 mRNA levels increased at postnatal days (P)14 and P30 with respect to the level at P7 both in liver and brain, this increment was especially pronounced in the brain at P14. The expression of CYP1A2 in the brainstem (BS) was higher than that in the cerebellum (CLL) and cortex (COR). Meanwhile, the CYP1A2 protein level was significantly higher in the COR than in the brainstem and CLL at P14. The levels of BR and its metabolites (m/z values 301, 315, 333 and biliverdin) were statistically unaltered by incubation with liver and brain microsomal fractions.

**Conclusion:** Our results indicated that the region-specific expression of CYP1A2 increased during development, but CYP family enzymes were physiologically incapable of metabolizing BR. The ability of CYPs to oxidize BR may be triggered by CYP inducers.

## Introduction

Bilirubin (BR), a potent antioxidant that protects neurons against oxidative-free radicals, is an important endogenous antioxidant in humans (Kapitulnik, 2004; Sticova and Jirsa, 2013). Serum BR has had been shown to have a protective effect against cardiovascular, metabolic disease (Hinds and Stec, 2018). BR’s redox activity is particularly important in the brain, where it prevents excitotoxicity and neuronal death (Vasavda et al., 2019). Recent studies also reported that BR can bind to activate PPARα transcriptional activity to suppress fat accumulation (Stec et al., 2016; Gordon et al., 2019). However, patients especially neonates with acute unconjugated hyperbilirubinemia, such as increased BR production and delayed maturation of the hepatic conjugation system or inherited genetic disorders, are at risk for BR encephalopathy. There is a noticeable regional topography in BR-induced central nervous system (CNS) injury (Watchko and Tiribelli, 2013). Increased BR primarily injures a subgroup of neurons in areas of the basal ganglia, brainstem, and CLL (Watchko, 2006; Watchko and Tiribelli, 2013). Differences in BR clearance and the potential role of BR-metabolizing enzymes are largely proposed as a reasonable explanation for this(Brodersen and Bartels, 1969; Hansen and Allen, 1996). BR is metabolized mainly *via* uridine diphosphate (UDP)-glucuronosyltransferase 1A1 (UGT1A1)-mediated glucuronidation in the liver (Ma et al., 2014). However, the regional specificity of BR toxicity can also be observed in the Gunn rat, a model of kernicterus due to a spontaneous mutation in the UGT1A1 gene (Watchko, 2006; Watchko and Tiribelli, 2013), indicating that UGT1A1 may be not the reason for the CNS injury topography.

Cytochrome P450 1A2 (CYP1A2), an important member of the CYP superfamily, is expressed in the liver and extrahepatic tissues, including the brain (Nelson et al., 1996). CYP1A2 is responsible for phase I oxidative reactions in the activation of aromatic and heterocyclic amines and numerous therapeutic drugs (Zhou et al., 2010). In general, CYP1A2 induction is a means of maintaining homeostasis of the chemical environment in cells by increasing the metabolic clearance of substrates (Gunes and Dahl, 2008). Several studies have suggested that CYP may be an alternative BR degradation enzyme based on observations that the induction of CYP1A2 may reduce blood plasma BR and increase biliary excretion of hydroxylated metabolites in normal and Gunn rats (Schmid and Hammaker, 1963; De Matteis et al., 1991; Kapitulnik and Gonzalez, 1993). Studies have shown that BR can be degraded through mitochondrial and microsomal CYPs in the brain (Hansen and Allen, 1996; Hansen et al., 1999). Moreover, the BR concentration was lower in regions where CYPs were highly induced, substantiating a possible role of CYPs in controlling local BR concentrations in the brain(Hansen et al., 1999; Gazzin et al., 2012). Inducers (e.g., 2,3,7,8-tetrachlorodibenzo-p-dioxin [TCDD] or CdCl_2_) were used to stimulate liver microsomal CYP to investigate its role in BR metabolism in these studies (De Matteis et al., 1989; Kapitulnik and Gonzalez, 1993; Abu-Bakar et al., 2005). To date, the effect of CYPs on BR metabolism has not been identified under physiological conditions in normal rats.

In this study, we investigated the expression of CYP1A2 in the brain and the liver and examined BR metabolites in microsomal fractions during development without using any inducers. The aim of this study was to clarify the role of CYP1A2 in the metabolism of BR during development under normal physiological conditions in different regions of the brain and liver. The results will be helpful in elucidating the physiological role of CYP in BR metabolism and BR-induced CNS injuries.

## Materials and Methods

### Materials and Chemicals

BR, biliverdin, Tween 20, Triton X-100, acetonitrile (high-performance liquid chromatography [HPLC] grade), dimethyl sulfoxide (DMSO), 2-methylbutane, β-nicotinamide adenine dinucleotide 2′-phosphate-reduced tetrasodium salt hydrate (NADPH), ethylenediaminetetracetic acid (EDTA), and dithiothreitol (DTT) were purchased from Sigma-Aldrich (Sydney, Australia). PrimeScript^™^ RT Master Mix kits were obtained from TaKaRa Bio, Inc. (Beijing, China). FastStart SYBR Green Master Mix kits were purchased from Roche Diagnostics (Indianapolis, IN, USA). The *in vitro* Cell-Free CYP1A2 Assay Kit was purchased from GenMed Scientifics, Inc. (Arlington, MA, USA). Furafylline and CYP1A2 antibodies were purchased from Abcam (Cambridge, UK). All chemicals and solvents were of analytical grade.

### Animals

Sprague-Dawley rats were housed in filter-top polycarbonate cages containing wood chip bedding and maintained at 24°C with a 12-h photoperiod and free access to standard rat chow and tap water. All procedures involving animal care or treatments were approved by and conducted in accordance with institutional guidelines and national and international laws and policies. This study was approved by the Ethics Review Committee for Animal Experimentation of Shanghai Jiaotong University. To minimize contamination of tissues with unconjugated bilirubin (UCB) in blood, the anesthetized animals were perfused with normal saline at 4°C through the cannulated left ventricle of the heart. The liver was used for RNA, protein, and microsome extraction. The whole brain was used for microsome extraction. The brainstem, CLL, and COR were isolated under a microscope and used for RNA and protein extraction. The meninges were removed from all brain tissues.

### Quantitative Real-Time Polymerase Chain Reaction

Quantitative real-time polymerase chain reaction (QRT-PCR) was conducted for analysis of CYP1A2 mRNA. Total RNA was isolated from the liver, brainstem, CLL, and COR using TRIzol^®^ reagent (TaKaRa Bio, Inc., Beijing, China). Total RNA purity and integrity were assessed by a NanoDrop 2000c (Thermo Fisher Scientific, USA). One microgram of total RNA was reverse transcribed with PrimeScript™ RT Master Mix (TaKaRa Bio, Inc., Beijing, China) according to the manufacturer’s instructions. QRT-PCR was performed in a 10-µL volume containing a 2-µL sample of cDNA, 2 µL of FastStart SYBR Green Master Mix (Roche Diagnostics, Indianapolis, IN, USA), and each primer at a final concentration of 0.4 µM. The amplification protocol was as follows: 3 min at 95°C followed by 40 cycles at 95°C for 5 s, 60°C for 30 s, and 72°C for 30 s. The mRNA levels were normalized against the mRNA levels of the housekeeping gene GAPDH. The amplicons from the QRT-PCR were verified by melting curve analysis and electrophoresis on a 1.5% agarose gel to ensure the absence of primer-dimer formation and the specificity of the amplicons and that a single band was detected at the expected size. Relative quantification was calculated using the ΔΔCt method (Livak and Schmittgen, 2001). The gene-specific primers for CYP1A2 and GAPDH are listed in **Table 1**.

**Table 1:** Gene specific primers used in RT-PCR.

Gene	Accession number	Forward	Reverse	Size
CYP1A2	NM_012541.3	GTGGTGGAATCGGTGGCTAATGTC	GGGCTGGGTTGGGCAGGTAG	175 bp
GAPDH	NM_017008	CTCTCTGCTCCTCCCTGTTC	CACCGACCTTCACCATCTTG	87 bp

### Preparation of Rat Microsomes and Western Blot Assay

The microsomes were prepared from rat liver and brain tissues using standard differential ultracentrifugation methods (Chahin et al., 2013). The tissues were homogenized in microsomal homogenization buffer (20 mM Tris-HCl, 250 mM sucrose, 1 mM EDTA, and 0.1 mM DTT; pH 7.4) and centrifuged at 10,000 g for 30°min at 4°C. The supernatant was centrifuged at 110,000 g for 70 min at 4°C using a Beckman LE-80K Optima centrifuge with a Type 70.1 Ti rotor (Beckman, Milan, Italy) (Zaccaro et al., 2001; Gambaro et al., 2014). Pellets were resuspended in microsomal storage buffer (20% glycerol, 20 mM Tris, 0.1 mM potassium phosphate, 0.1 mM DTT, and 1 mM EDTA; pH 7.4, 4°C). The microsomes were stored at −80°C until further use.

Protein concentrations in the liver, brainstem, CLL, and COR were determined using a bicinchoninic acid protein assay (BCA) kit (CW Biotech, Shanghai, China) according to the manufacturer’s instructions. Protein (35 μg) fractions were separated by a NuPAGE^®^ 10% w/v Bis-Tris 1.5 mm mini-gel (Invitrogen, Australia), transferred onto a polyvinylidene difluoride (PVDF) membrane and blocked for 2 h in Tris-buffered saline containing 0.1% Tween 20 (TBST) and 5% nonfat milk. Blots were incubated with a mouse monoclonal anti-CYP1A2 antibody (Abcam, Cambridge, UK) at a 1:1000 dilution overnight at 4°C and washed with TBST. The membranes were then incubated with a horseradish peroxidase (HRP)-conjugated goat antimouse antibody (Beyotime Biotechnology, Shanghai, China) at a 1:2000 dilution for 2 h and washed further with TBST. Protein bands were detected with ECL Plus Western Blotting Substrate (Beyotime, Shanghai, China). Blots were scanned with an HP Scanjet 5530 (Hewlett-Packard, Palo Alto, California, USA), and the intensity of each band was quantitated using the NIH ImageJ software (Frederick, MD, USA). The densitometric values of the bands were normalized to those of GAPDH.

### Immunofluorescence and Confocal Microscopy

Livers were obtained from 4% paraformaldehyde (PFA)-perfused rats. The tissues were cryoprotected in 15% and 30% sucrose in PBS for 12 h at 4°C and then frozen in liquid nitrogen. Brain tissues from the normal saline-perfused rats were immediately snap-frozen in isopentane for 10 s (Nazarali et al., 1987; Roos et al., 1997). Twenty-micrometer-thick sections were prepared and permeabilized in 4% PFA for 15 min. After PBS washes, the slides were incubated with blocking buffer (PBS with 0.5% Triton X-100 and 5% normal goat serum) for 2 h at room temperature. CYP1A2 primary antibodies (Abcam, Cambridge, UK) were diluted 1:800 in blocking buffer and added to the slides for incubation overnight at 4°C in a humidified chamber, and the slides were then washed with 0.1% Triton X-100 in PBS. For visualization, the slides were incubated with an Alexa Fluor^®^ 488-labeled antimouse secondary antibody (Eugene, Oregon, USA) and nuclei were counterstained with 4′,6-diamidino-2-phenylindole (DAPI). The localization of CYP1A2 in the brain regions was determined from the immunofluorescence staining. Images were acquired using a confocal microscope (Zeiss 710, Jena, Germany).

### Enzyme Activity Assay

CYP1A2 enzyme activity was assessed using the 7-methoxyresorufin-O-demethylase (MROD) Activity Kit (GenMed Scientifics, Inc., Arlington, MA, USA) according to the manufacturer’s instructions. MROD activity was measured spectrofluorometrically. The excitation/emission wavelengths were 535 and 590 nm, respectively.

### Bilirubin Oxidation

The BR disappearance assay was performed as previously described with a slight modification (Abu-Bakar et al., 2005). Briefly, incubation buffer (100 mM Tris-HCl (pH 8.2), 2 mM MgCl2, and 2 mM EDTA) was mixed with extracted microsomal fractions (50 μg total protein/100 μL) in a total volume of 200 μL. After 5-min preincubation, NADPH (final concentration = 1 mM) was added to start the reaction. BR in DMSO (5 μM final concentration) was immediately added to the sample plate, and the absorbance at 440 nm was recorded for 15 cycles at 1 min/cycle. The rate of BR disappearance was determined with a PerkinElmer Envision multilabel plate reader (PerkinElmer, Milan, Italy) and expressed as pmol BR disappearing/min/nmol P450.

### HPLC/Electrospray Ionization Tandem Mass Spectrometry (ESI-MS)

BR metabolites in the isolated liver and brain microsomal fractions were identified using high-performance liquid chromatography/electrospray ionization tandem mass spectrometry (LC-MS/MS). A rapid and sensitive LC-MS/MS method was established for semiquantification of BR and its active metabolites (Abu-Bakar et al., 2011; Abu-Bakar et al., 2012). Five groups were subjected to experimental paradigms as described in **Table S1**. Briefly, the mixture was incubated at 37°C for 40 min in the dark. Acetonitrile (600 μL) was added to the samples to stop the reaction, and the mixture was centrifuged. The supernatants were injected into the liquid chromatography mass spectrometer (LC-MS). The LC-MS/MS system was equipped with an Agilent 1200 liquid chromatograph and a 6410B triple quadrupole mass spectrometer with an ESI source. Data were analyzed using the MassHunter software (Agilent Corporation, Lexington, MA, USA). Chromatography was performed on a Phenomenex 00B-4372-B0 HPLC column (2*50 mm, 2 µm; Torrance, CA, USA) maintained at 35ºC. The mobile phase consisted of 5 mM ammonium formate and 0.1% formic acid in purified water (Solvent A) and acetonitrile (Solvent B). The gradient program was as follows: 0 min, 20% B; 1 min, 90% B; 6 min, 90% B; 7 min, 20% B; and 12 min, 20% B. The flow rate was set at 0.35 mL/min. ESI was performed in the positive ion mode, and molecules were quantified by the selected reaction monitoring transitions of the protonated molecular ions at the following mass-to-charge ratios (m/z): BR at 585.3−299.2, biliverdin at 583.3−297.1, M3 at 333, M2 at 315, M1 at 301.1, and donepezil-d5 (internal standard) at 385.3−96.1. Donepezil as internal standards in our study is to correct the instrument variation of ion suppression or enhancement in detection. Both the nebulizer and the drying gases were high-purity nitrogen, and the gas temperature and gas flow were set at 350°C and 10.0 L/min. Other ESI source conditions were as follows: nebulizer pressure, 40 psi; capillary voltage, 4,000 V; dwell time, 200 ms, and EMV (electron multiplication voltage), 200 V.

### Statistical Analysis

Data were analyzed with GraphPad Prism^®^ 5 for Windows. Two-group comparisons were made using Student’s t-tests. Multiple-group comparisons were accomplished with a one-way analysis of variance (ANOVA), followed by Bonferroni’s *post hoc* test to determine significance between experimental groups with one independent variable. The results from one group were compared using the nonparametric Mann-Whitney U test. All data are shown as the means ± standard deviation (SD). A probability level of P < 0.05 was considered statistically significant.

## Results

### CYP1A2 mRNA Expression in the Liver and Selected Brain Regions During Development

**Figure 1:**
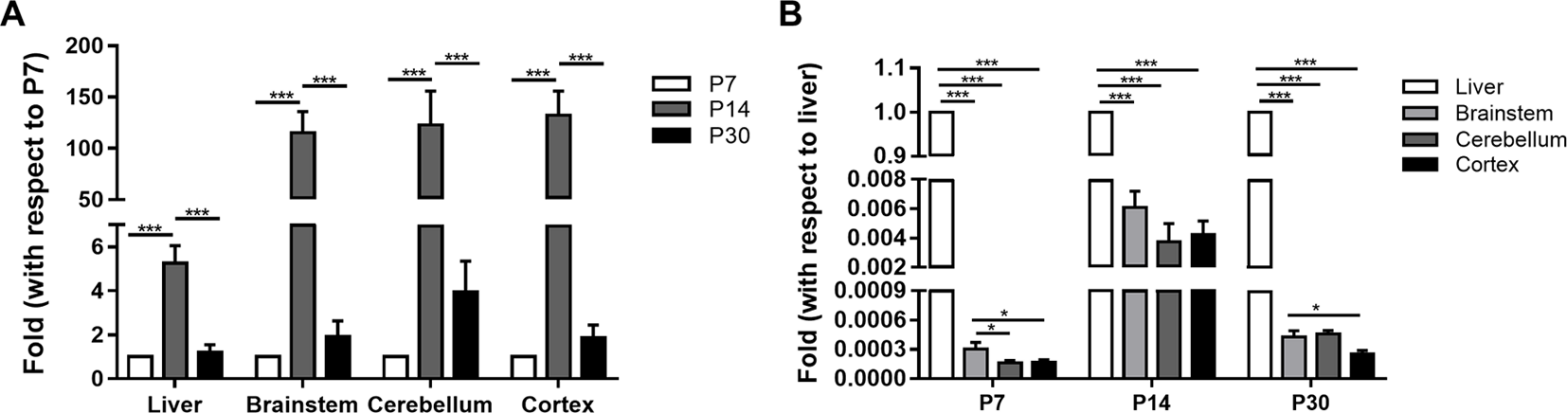
The relative expression of CYP1A2 mRNA in the liver and brain. **(A)** The fold changes of CYP1A2 mRNA expression with respect to 7-day-old SD rats. **(B)** The fold changes of CYP1A2 mRNA expression with respect to liver. GAPDH was used for normalization. Data are expressed as the means ± standard deviation (SD) of five to six biological replicates. *p < 0.05, ***p < 0.001. P, postnatal day.

The calibration analysis including slope and R2 derived from the standard curve of CYP1A2 real time RT-PCR and melt curve were −3.46 and 0.997, and GAPDH were −3.32 and 0.998, respectively. CYP1A2 mRNA was expressed in a region-specific and age-dependent manner in both the liver and brain (**Figure 1**). The CYP1A2 mRNA levels peaked at P14 and dropped several times to their P30 values in both the liver and the brain. In the liver, BS, CLL, and COR, a strong increase in the CYP1A2 mRNA levels (fold changes of 5.3 ± 0.8, 115.4 ± 20.5, 122.9 ± 32.9, and 132.3 ± 13.4, respectively) was observed at P14 compared with the levels at P7. At P30, the CYP1A2 mRNA levels exhibited fold changes of 1.2 ± 0.3, 1.9 ± 0.7, 4.0 ± 1.4, and 1.9 ± 0.6 in the liver, BS, CLL, and COR, respectively, with respect to their levels at P7 (**Table S2**). The CYP1A2 mRNA levels in the liver were much higher than those in the brainstem, CLL, and COR (P < 0.001). The levels of CYP1A2 mRNAs in the BS were higher than those in the CLL and COR (**Table S3**).

### CYP1A2 Protein Expression in the Liver and Brain

**Figure 2:**
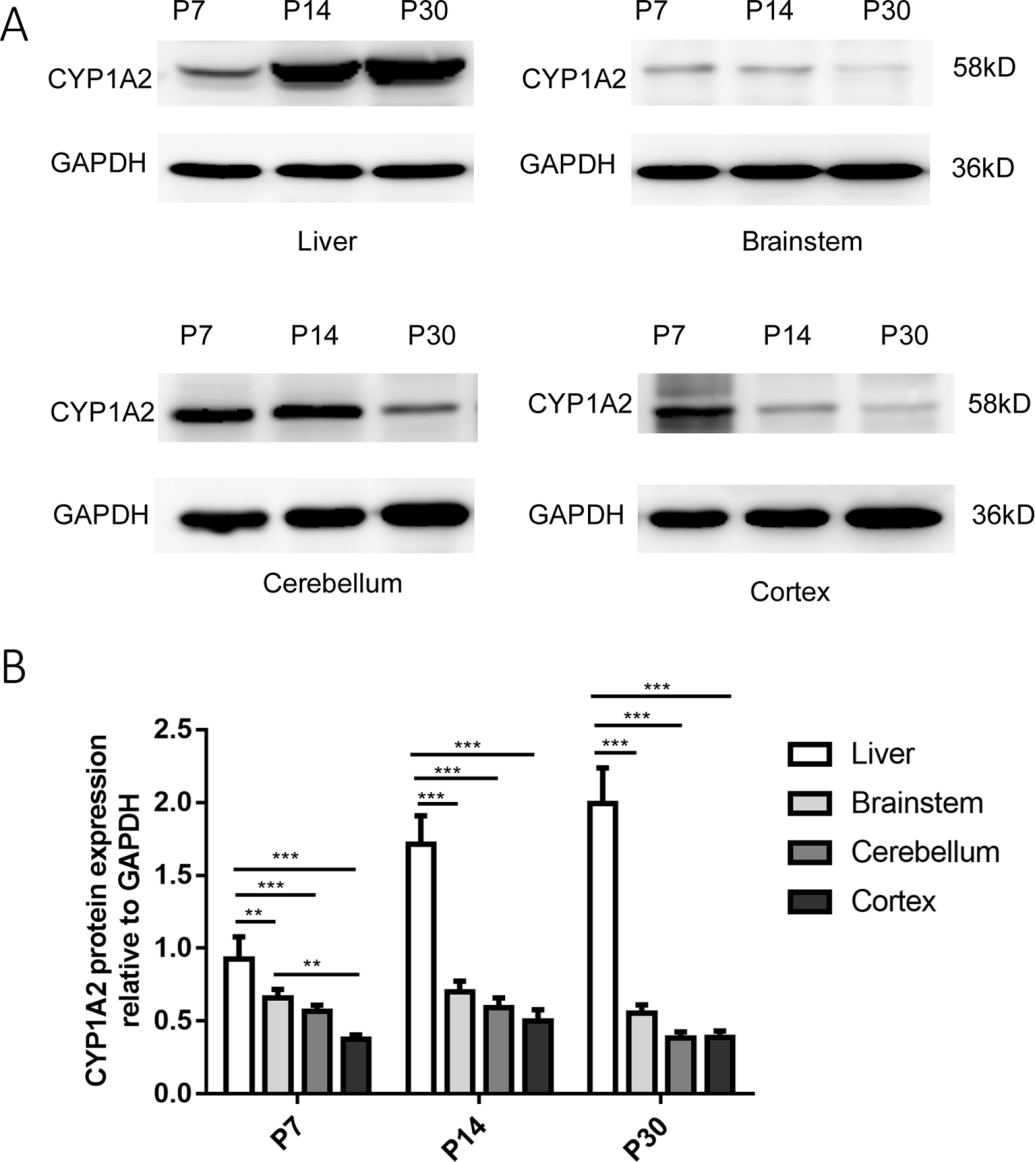
Expression of CYP1A2 protein in the liver and brain. **(A)** Representative western blot and relative protein expression in the liver and different regions of the brain. The bands at 58 and 36 kDa correspond to CYP1A2 and GAPDH, respectively. **(B)** GAPDH protein levels are shown as a control. Expression values of protein levels were normalized to those of GAPDH. Each data point represents the mean ± SD of six samples **p < 0.01, ***p < 0.001. P, postnatal day.

We determined that the CYP1A2 protein was expressed in the liver in a development-dependent manner by western blot analysis; this finding was consistent with the mRNA expression data (**Figure 2**). CYP1A2 protein expression was significantly higher in the liver than in the brain. No significant changes in protein levels were observed in the brainstem, CLL, or COR during development. There were no differences among the three regions at P14 and P30. However, at P7, the band intensity of CYP1A2 protein in the brainstem was nearly 1.75-fold that in the COR. These results indicate that protein levels of CYP1A2 in the liver increased with age. Furthermore, there were no regional differences in CYP1A2 protein expression in brain tissue.

### CYP1A2 in the Brain Regions At the Three Developmental Stages

The pontine nucleus (PN), ventral cochlear nucleus (VCN), medial vestibular nucleus (MVE), CLL, and COR were examined using immunofluorescence during development. These nuclei are more vulnerable to BR toxicity than other brain locations. Neuronal cells in the CNS were generally well stained in the brainstem, CLL, and COR. Green fluorescence was observed in the cell cytoplasm. It appeared that all aspects of neurons visible on the COR surface were somewhat more stained than other nuclei, but the staining could not be considered extremely specific. We also observed more green fluorescence staining at P30, but no specialization was noted.

**Figure 3:**
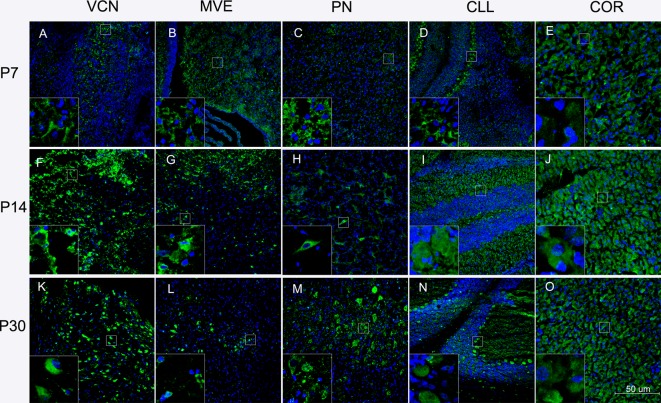
Localization of CYP1A2 in the brain regions at different ages. **(A)** Localization of CYP1A2 in the VCN at P7. **(B)** Localization of CYP1A2 in the MVE at P7. **(C)** Localization of CYP1A2 in the PN at P7. **(D)** Localization of CYP1A2 in the CLL at P7. **(E)** Localization of CYP1A2 in the COR at P7. **(F)** Localization of CYP1A2 in the VCN at P14. **(G)** Localization of CYP1A2 in the MVE at P14. **(H)** Localization of CYP1A2 in the PN at P14. **(I)** Localization of CYP1A2 in the CLL at P14. **(J)** Localization of CYP1A2 in the COR at P14. **(K)** Localization of CYP1A2 in the VCN at P30. **(L)** Localization of CYP1A2 in the MVE at P30. **(M)** Localization of CYP1A2 in the PN at P30. **(N)** Localization of CYP1A2 in the CLL at P30. **(O)** Localization of CYP1A2 in the COR at P30. P, postnatal day. PN, pontine nucleus; VCN, ventral cochlear nucleus; MVE, medial vestibular nucleus; CLL, cerebellum; COR, cortex.

To our knowledge, this study is the first to detect CYP1A2 immunoreactivity in neuronal cell nuclei in the brain. In the brain, the fluorescence intensity of some nuclear areas was not identical, suggesting CYP1A2 nuclear specificity in the brain. Representative images of CYP1A2 localization are shown in **Figure 3**.

### Bilirubin and Its Metabolites

**Figure 4:**
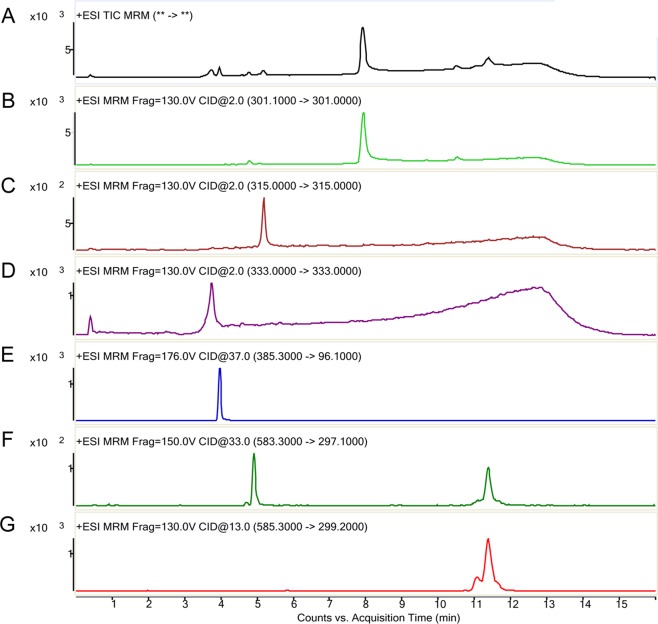
Representative LC-MS/MS chromatographs of bilirubin and its metabolites (counts vs. acquisition time). The various ions detected are indicted in the order of their elution time. **(A)** Total ion current; **(B)** Reaction monitoring transitions of M/Z 301; **(C)** Reaction monitoring transitions of M/Z 315; **(D)** Reaction monitoring transitions of M/Z 333; **(E)** Multiple-reaction monitoring of M/Z 385→96.1 (donepezil, internal standard); **(F)** multiple-reaction monitoring of biliverdin M/Z 583→297; **(G)** multiple-reaction monitoring of bilirubin M/Z 585→299.

The MROD of CYP1A2 enzyme activity was 8.1–98.9 nmol/min. The rates of microsomal BR degradation are shown in **Table 2**. The liver microsomes degrade BR significantly faster (by 10-fold) than those of the brain. Representative chromatograms for BR and its metabolites are shown in **Figure 4**. Total ion current (**Figure 4A**), donepezil-d5 (m/z 385, **Figure 4E**), biliverdin (m/z 583, **Figure 4F**), BR (m/z 301, **Figure 4G**), and the active metabolites BM1 (m/z 301, **Figure 4B**), BM2 (m/z 315, **Figure 4C**), and BM3 (m/z 333, **Figure 4D**) were identified by determining their retention times and molecular weights in this analysis. The retention times of the metabolites and the relative molecular intensity of liver and brain microsomal BR degradation are shown in **Tables 3** and **4**, respectively. There is no significant difference among these groups.

**Table 2:** Bilirubin degradation rate of microsomes.

Microsome fractions	Rate of bilirubin degradation*
Liver	0.68 ± 0.005
Brain	0.07 ± 0.018

*: pmol BR disappearing/min/nmol P450.

**Table 3:** Analytical data counts and acquisition time of liver incubation systems.

Ion(M/Z value)	Retention time (min)	BR no incubation	BR incubation	BR+Liver microsome	BR+Liver microsome + NADPH	BR+Liver microsome + NADPH + furafylline	P
301*	7.937 ± 0.007	15.982 ± 1.237	17.809 ± 0.962	15.881 ± 0.635	16.811 ± 1.500	16.444 ± 0.672	0.144
	12.570 ± 0.154	17.112 ± 4.680	17.779 ± 4.310	15.290 ± 1.060	13.068 ± 1.425	12.426 ± 1.423	0.131
315*	5.182 ± 0.005	0.848 ± 0.093	0.810 ± 0.040	0.770 ± 0.083	0.782 ± 0.115	0.813 ± 0.076	0.791
	12.618 ± 0.143	2.581 ± 0.007	2.844 ± 0.348	3.722 ± 2.333	2.138 ± 0.383	4.059 ± 1.640	0.315
333*	0.422 ± 0.002	5.230 ± 0.454	5.830 ± 0.278	4.746 ± 0.052#	0	0	0.000
	12.731 ± 0.084	18.208 ± 0.960	17.052 ± 1.450	24.690 ± 9.360	16.700 ± 0.970	15.912 ± 0.989	0.120
Bilirubin*	11.383 ± 0.009	2.471 ± 0.430	2.117 ± 0.354	1.820 ± 1.119	2.055 ± 0.291	2.165 ± 0.883	0.844
	11.070 ± 0.030	1.409 ± 0.154	1.015 ± 0.376	0.400 ± 0.200 &#	0.510± 0.082 &	0.565 ± 0.100 &	0.000
Biliverdin*	11.383 ± 0.008	0.094± 0.018	0.066 ± 0.014	0.055 ± 0.029	0.095 ± 0.017	0.111 ± 0.028	0.141
	4.908 ± 0.007	0.062 ± 0.018	0.052 ± 0.013	0.068 ± 0.048	0.098 ± 0.009	0.088 ± 0.019	0.035

The retention time and relative molecular intensity of liver microsomes. There is no statistical difference among these groups.

*: area of BR/area of IS; IS, donepezil (DNP, internal standard); BR, bilirubin

**Table 4:** Analytical data counts and acquisition time of brain incubation systems.

Ion(M/Z value)	Retentiontime (min)	BR no incubation	BR incubation	BR+Brain microsome	BR+Brain microsome +NADPH	BR+Brain microsome + NADPH + furafylline	P
301*	7.937 ± 0.007	15.982 ± 1.237	17.809 ± 0.962	18.404 ± 3.383	16.264 ± 3.745	19.493 ± 4.904	0.643
	12.570 ± 0.154	17.112 ± 4.680	17.779 ± 4.310	15.561 ± 2.058	17.108 ± 1.257	16.017 ± 4.505	0.942
315*	5.182 ± 0.005	0.848 ± 0.093	0.810 ± 0.040	0.842 ± 0.062	0.832 ± 0.042	0.0842 ± 0.060	0.915
	12.618 ± 0.143	2.581 ± 0.007	2.844 ± 0.348	2.585 ± 0.069	2.728 ± 0.112	2.997 ± 1.142	0.897
333*	0.422 ± 0.002	5.230 ± 0.454	5.830 ± 0.278	5.234 ± 0.372	0	0	0.000
	12.731 ± 0.084	18.208 ± 0.960	17.052 ± 1.450	24.652 ± 11.957	17.005 ± 0.522	17.138 ± 2.554	0.382
Bilirubin*	11.383 ± 0.009	2.471 ± 0.430	2.117 ± 0.354	1.949 ± 0.584	2.270 ± 0.703	2.976 ± 0.668	0.140
	11.070 ± 0.030	1.409 ± 0.154	1.015 ± 0.376	0.533 ± 0.098#	0.581 ± 0.034#	0.557 ± 0.174#	0.001
Biliverdin*	11.383 ± 0.008	0.094± 0.018	0.066 ± 0.014	0.067 ± 0.019	0.092 ± 0.017	0.121 ± 0.028	0.014
	4.908 ± 0.007	0.062 ± 0.018	0.052 ± 0.013	0.057 ± 0.020	0.085 ± 0.021	0.094 ± 0.037	0.148

The retention time and relative molecular intensity of brain microsomes. There is no statistical difference among these groups.

*: area of BR/area of IS; IS, donepezil (DNP, internal standard); BR, bilirubin

## Discussion

In humans, BR glucuronidation is mainly catalyzed by hepatic UGT1A1. It has been reported that an alternative pathway of oxidative degradation of BR becomes activated in neonatal jaundice and in hereditary forms of congenital jaundice, where the main glucuronidation pathway is defective (Kapitulnik and Ostrow, 1978; Kaul et al., 1980). Previous *in vitro* and *in vivo* findings on CYP450-mediated oxidative degradation of BR obtained from rats and mice treated with powerful CYP1A2 inducers, such as TCDD, b-naphthoflavone (β-BNF), or 3-methylcholanthrene (3-MC), strongly indicated that CYP1A2 activity contributes significantly to the metabolism of BR in Gunn rats (Cohen et al., 1986; Kapitulnik and Gonzalez, 1993; Zaccaro et al., 2001; Gambaro et al., 2014). However, it is critical to clarify the metabolic role of CYP under normal physiological conditions.

The present study detected CYP1A2 mRNA and protein in different brain regions and in the liver. The results showed that CYP1A2 mRNA and protein levels were significantly higher in the liver than in the brain regions, which agreed with the results of the previous study (Ferguson and Tyndale, 2011). In addition, this is the first study to quantitate the expressions of CYP1A2 mRNA in the brain and the liver. The green fluorescence staining detected in the nuclear regions in our study further demonstrated the distribution of the enzyme in the brain. The region-specific expression of CYP1A2 mRNA in the brain may provide some insight into its functional significance and metabolic roles. The role of CYP1A2 in BR degradation had been studied many years. Regional distribution of this protein were also investigated in human and adult rodents brain (Farin and Omiecinski, 1993; McFadyen et al., 1998; Morse et al., 1998; Dey et al., 1999).

Considering the potential ability of CYP1A2 to degrade various compounds and drugs, the ability of CYP1A2 to metabolize BR under physiological conditions should not be ignored (Kapelyukh and Henderson, 2019). Metabolite screening by LC/MS-MS showed that the levels of BR and its metabolites, biliverdin, BM1 (m/z 301), BM2 (m/z 315), and BM3 (m/z 333), did not significantly change with treatment. These results indicated that CYPs were incapable of metabolizing BR in both brain and liver microsomes in Sprague-Dawley (SD) rats. Under physiological condition, the rate of BR degradation is no significant difference between wild type and Cyp1a2(2/2) mutant mice (Zaccaro et al., 2001), and and Cyp1 or Cyp2 knockout animals do not develop elevated blood BR levels (Fujiwara et al., 2018). Meanwhile, BR is partly converted to oxidized metabolites by cytochrome P450 (CYP) 1 and CYP2 family enzymes (Fujiwara et al., 2018).

De Matteisa and coworkers concluded that CYP1A2 is responsible for microsomal BR degradation in the absence of 3,4,3′,4′-tetrachlorobiphenyl (TCB) as an inducer and that TCB was also required for BR degradation by CYP1A1 (Zaccaro et al., 2001). Gazzin also reported that cyp1A1 can oxidize BR only after uncoupling by TCB and that Cyp1A2 can be active in BR clearance without uncoupling in primary astrocytes (Gambaro et al., 2014). These studies concentrated on BR degradation with induction or in Gunn rats.

Several inducers were chosen to selectively induce upregulation of the enzyme in previous studies focusing on the role of CYP1A2 in BR oxidation (Zaccaro et al., 2001; Gambaro et al., 2014). It has also been reported that CYP1A2 mRNA was induced to different degrees in various brain regions in a dose-dependent manner (Gazzin et al., 2012). The mRNA and protein levels of CYP1A2 were also markedly elevated in jaundiced rats. The induced CYP1A2 is responsible for degrading BR. However, the exact mechanism of CYP-catalyzed BR metabolism (e.g., in UGT1A1 deficiency) in animals treated with inducers or in Gunn rats still requires clarification. A previous study proposed that TCDD and similar inducers bind the active site of the induced CYP as poor substrates, so that electron flow from NADPH to oxygen is facilitated and partially reduced oxygen species are produced in excess, which may be responsible for the observed oxidation of BR (De Matteis et al., 2012). In that case, CYP1A2 would act mainly as a binding trap and would be eliminated subsequently by ubiquitinylation (Fukai et al., 1989). This process may contribute to the negative result for CYP metabolism of BR observed in this study.

Both *in vivo* and *in vitro* experiments have shown that CYP1A2 levels were more highly induced in the COR than in the CLL, where UCB accumulation was more consistent (Iba et al., 2003; Gazzin et al., 2012; Gambaro et al., 2014). Notably, this pattern paralleled the topography of BR neurotoxicity, which showed that the CLL was the major landmark of BR toxicity in animal models of hyperbilirubinemia, whereas no damage was observed in the cerebral COR (Schutta and Johnson, 1967; Gazzin et al., 2012; Gambaro et al., 2014). These results clearly indicated that the regionally selective induction of CYP1A2, together with its differential catalytic ability to oxidize BR, may explain the lower sensitivity to BR toxicity of the COR compared with the CLL. Notably, BR is not only a CYP substrate but also a CYP inducer (Abu-Bakar et al., 2012). A previous study showed that CYP mRNA was more highly elevated in the cerebral COR (resistant region) than in the CLL (damaged region) in Gunn rats after an acute BR load (Gazzin et al., 2012). The differential degree of CYP induction by inducers (including BR itself) in the various brain regions may underlie the differential ability of CYPs to oxidize BR and the mechanism of the regional topography of BR neurotoxicity. Recently, a couple of studies discovered the role of BR as a hormone, which can bind to activate PPAR-α transcriptional activity. Therefore, it is possible that BR is inducing PPAR-α transcriptional activity in brain to regulate the CYP enzymes. This may also explain differences in response in patients if some patients do not have this receptor expressed in the brain region (Stec et al., 2016; Gordon et al., 2019). Hansen’ s group reported that the BR oxidizing activity in brain mitochondria was dependent on cytochrome c, but not on NADPH (Hansen and Allen, 1996; Hansen and Allen, 1997; Hansen et al., 1999). It suggested that mitochondria may play a more important role in the metabolism of BR in the brain.

In the present study, we tried to find expression of CYP1A2 in the brain and the liver and examined BR metabolites in microsomal fractions during development. Understanding systemic regulation of BR during development may be helpful for corresponding toxicological treatment. However, there is not available data about BR metabolites in microsomal fractions during development. In addition, the compensatory role of other cytochrome P-450 enzymes should also be observed.

In conclusion, our work demonstrated that CYP1A2 is enriched in the liver and widely distributed in the rat brain, but it is incapable of metabolizing BR under physiological conditions. We propose that the ability of CYP to metabolize BR is triggered by CYP inducers (including BR itself). The regionally selective induction of CYP1A2, together with its differential catalytic ability to oxidize BR, may underlie the topographical mechanism of BR neurotoxicity.

## Data Availability Statement

All datasets generated for this study are included in the manuscript/**Supplementary Files**.

## Ethics Statement

All experiments were approved by the Ethics Review Committee for Animal Experimentation of Shanghai Jiaotong University.

## Author Contributions

SY, DY, and HS conceived and designed the study. XL, CL, HJ, HZ, HY, GM, and LW performed the experiments and procedure. XL and CL wrote the manuscript.

## Funding

This study was supported in part by the State Key Development Program for Basic Research of China (2014CB541705), the National Natural Science Foundation of China (81470688), the National Natural Science Foundation of China (8140030831), the Excellent Personnel Training Plan for the Shanghai Health System (grant number 2017YQ010), the National Natural Science Foundation of China (81470690), the Major Program of Shanghai Committee of Science and Technology (14DJ1400202), and International Joint Projection of Science and Technology Commission of Shanghai Municipality (14430720900).

## Conflict of Interest

The authors declare that the research was conducted in the absence of any commercial or financial relationships that could be construed as a potential conflict of interest.

## Abbreviations

BR, bilirubin; BS, brainstem; CNS, central nervous system; CLL, cerebellum; COR, cortex; CYP, cytochrome P450; DTT, dithiothreitol; EDTA, ethylenediaminetetracetic acid; HPLC, high-performance liquid chromatography; LC-MS/MS, high-performance liquid chromatography/electrospray ionization tandem mass spectrometry; MROD, 7-methoxyresorufin-O-demethylase; MVE, medial vestibular nucleus; NADPH, β-nicotinamide adenine dinucleotide 2′-phosphate-reduced tetrasodium salt hydrate; PFA, paraformaldehyde; PN, pontine nucleus; P, postnatal days; QRT-PCR, quantitative real-time polymerase chain reaction; UCB, unconjugated bilirubin; UDP, uridine diphosphate; UGT1A1, UDP-glucuronosyltransferase 1A1; VCN, ventral cochlear nucleus; TCDD, 2,3,7,8-tetrachlorodibenzo-p-dioxin.
