# Supplementary material for: Cytochrome P450 1A2 Is Incapable of Oxidizing Bilirubin Under Physiological Conditions
Source: Front Pharmacol. 2019 Oct 18;10:1220. doi: 10.3389/fphar.2019.01220 (PMC6813656; doi:10.3389/fphar.2019.01220)
Supplement: Supplementary file 1 [file Table_1.docx]

**Supplementary materials**

Table S1. Microsomes incubation system （*n*=5）

| group | BR | Oxidation buffer | microsome | NADPH | furafylline |
| --- | --- | --- | --- | --- | --- |
| 1 | √(no incubation) | √ |  |  |  |
| 2 | √ | √ |  |  |  |
| 3 | √ | √ | √ |  |  |
| 4 | √ | √ | √ | √ |  |
| 5 | √ | √ | √ | √ | √ |

Table S2. The folds of CYP1A2 mRNA expression with respect to P7

| Folds | Liver | Brainstem | Cerebellum | Cortex |
| --- | --- | --- | --- | --- |
| P14/P7 | 5.3±0.8 | 115.4±20.5 | 122.9±32.9 | 132.3±13.4 |
| P30/P7 | 1.2±0.3 | 1.9±0.7 | 4.0±1.4 | 1.9±0.6 |

P7: 7-day SD rat P14: 14-day SD rat P30: 30-day SD rat

Table S3. The folds of CYP1A2 mRNA expression with respect to liver

|  | P7 | P14 | P30 |
| --- | --- | --- | --- |
| Liver/BS | 4263±778 | 199±44 | 2672±339 |
| Liver/CLL | 7285±969 | 426±133 | 2261±143 |
| Liver/COR | 7153±1143 | 327±100 | 4614±750 |

BS: brainstem CLL: cerebellum COR: cortex
